# Strategies for achieving a high response rate in a home interview survey

**DOI:** 10.1186/1471-2288-9-46

**Published:** 2009-06-30

**Authors:** Kirsty Kiezebrink, Iain K Crombie, Linda Irvine, Vivien Swanson, Kevin Power, Wendy L Wrieden, Peter W Slane

**Affiliations:** 1 Division of Health and Food Sciences, School of Contemporary Sciences University of Abertay, Bell Street, Dundee, UK; 2 Department of Public Health, University of Dundee, Mackenzie Building, Kirsty Semple Way, Dundee, UK; 3 Department of Psychology, University of Stirling, Stirling, UK; 4 Department of Clinical Psychology, Dudhope Terrace, Dundee, UK; 5 Health Services Research Unit, University of Aberdeen, Health Sciences Building, Aberdeen, UK; 6 Erskine Practice, Arthurstone Medical Centre, Dundee, UK

## Abstract

**Background:**

Response rates in surveys have been falling over the last 20 years, leading to the need for novel approaches to enhance recruitment. This study describes strategies used to maximise recruitment to a home interview survey of mothers with young children living in areas of high deprivation.

**Methods:**

Mothers of two year old children received a letter from their GP inviting them to take part in a survey on diet. Participants were subsequently recruited by a researcher. The researcher first tried to contact potential participants by telephone, to discuss the study and make an appointment to conduct a home interview. Where telephone numbers for women could not be obtained from GP records, web searches of publicly available databases were conducted. After obtaining correct telephone numbers, up to six attempts were made to establish contact by telephone. If this was unsuccessful, a postal request for telephone contact was made. Where no telephone contact was achieved, the researcher sent up to two appointments by post to conduct a home interview.

**Results:**

Participating GPs invited 372 women to take part in a home based interview study. GP practices provided telephone numbers for 162 women, of which 134 were valid numbers. The researcher identified a further 187 numbers from electronic directories. Further searches of GP records by practice staff yielded another 38 telephone numbers. Thus, telephone numbers were obtained for 99% of potential participants.

The recruitment rate from telephone contacts was 77%. Most of the gain was achieved within four calls. For the remaining women, contact by post and home visits resulted in 18 further interviews, corresponding to 35% of the women not recruited by telephone. The final interview rate was 82%. This was possible because personal contact was established with 95% of potential participants.

**Conclusion:**

This study achieved a high response rate in a hard to reach group. This was mainly achieved by first establishing contact by telephone. The use of multiple sources identified the telephone numbers of almost all the sample. Multiple attempts at telephone contact followed by postal approaches led to a high home interview rate.

## Background

Concern with survey non-response and the potential for introducing bias is long standing [1]. These concerns are increasing because of evidence that non-response rates in many countries have risen over time [2]. An important distinction is made between refusal to participate and failure to establish contact with potential participants [3]. There is evidence that non-contact makes an important contribution to overall non-response [4] and that non-contact can result in substantial bias [5].

Considerable effort is often made to reduce non-response by making multiple attempts at contact through repeated calling, incentives to interviewers and respondents and introductory telephone calls. Systematic reviews of postal questionnaire surveys have shown that response rates are increased by hand signed letters [6], repeated mailings and telephone contact [7,8]. Similar strategies have been effective in recruiting subjects to randomised controlled trials [9,10]. Recent reviews have found that the provision of incentives is also effective [8,11,12]. The key requirement for all such approaches is that accurate contact details are available.

Low response rates are commonly associated with social deprivation [4,13,14] leading to a call for strategies to improve participation by disadvantaged groups [15]. Telephone contact has been found to improve socio-demographic representativeness [16]. Thus in a study of mothers with young children who lived in areas of high deprivation we made extensive efforts to contact potential participants. The main strategies were to ensure that accurate contact details were identified and to use telephone calls as the primary means of contact. This paper presents the techniques used and the results of these efforts.

## Methods

The dietary survey was designed to investigate maternal factors associated with poor diet among children living in areas of high deprivation. Families were suitable for inclusion if the mother lived with a two year old child and was responsible for buying, planning and providing the child's meals. Mothers were interviewed in their own homes using a computer aided personal interview (CAPI) which took approximately one hour to administer. The structured questionnaire included an assessment of the child's current diet, mother's knowledge about diet and cooking, her beliefs and attitudes to providing a healthy diet, and barriers to providing a healthy diet, such as access to shops of lack of cooking facilities.

Ethical permission for the dietary study was obtained from the local Committees on Medical Research Ethics in Tayside and Fife (Scotland) (Project reference number 04/S1401115). The Scottish Index of Multiple Deprivation (SIMD) [17] was used to identify areas of high deprivation. Ten GP practices located in the two most deprived deciles of Dundee and Fife were recruited. These practices supplied anonymised lists of all children aged two years who lived with their mother. From these lists the mothers with addresses in the two most deprived deciles of SIMD scores were identified. The UK Data Protection Act (1998) does not allow researchers direct access to patients' personal details. Thus, GPs checked patients' suitability for participation, then sent a letter of invitation together with an information leaflet about the study. The letter stated that if mothers did not wish to take part they should return an enclosed postcard, otherwise the research team would be given their contact details and a named researcher would contact them.

Following the letter of invitation from the GP, potential participants were contacted by a researcher who discussed the study and, if mothers were willing to take part, made an appointment to interview them in their own homes. As telephone call was the intended first means of contact by the researcher, several strategies were used to obtain telephone numbers. Internet searches of four databases in the public domain were used to obtain land line numbers [18,19] and mobile numbers [20]. The searches used combinations of the mother's surname, the child's surname if it differed from the mother's and parts of the address.

Up to six attempts were made to contact the women by telephone. When a woman refused to participate no further contact attempts were made. The timing of calls was varied to maximise the chances of contact: the first two calls were at different times of the working day, with subsequent calls in the evening and then at the weekend. Voicemail messages were left when it was clear that the telephone of the correct person had been contacted (ie a recorded message gave the person's name).

If no contact was established by telephone a letter was sent asking the women to contact the researcher. Women were asked to return a stamped response slip or telephone a designated number. Women who did not respond to this letter were then offered up to two appointments for home interviews at a specified time (one delivered by post, one by a hand-delivered visiting card).

Participants were given a €10 high street gift voucher [21] to thank them for their participation. The gift voucher was not mentioned at any of the contact attempts, but was offered after the interview was completed.

## Results

Participating GPs invited 372 women to take part in the study. Only three women opted out on receiving the letter of invitation from the GP. The home address of 36 women proved inaccurate, but after checking with GP staff this was reduced to six women with unknown addresses. Women whose current address could not be identified were excluded from further contact attempts. This left 363 women to be contacted.

**Figure 1:**
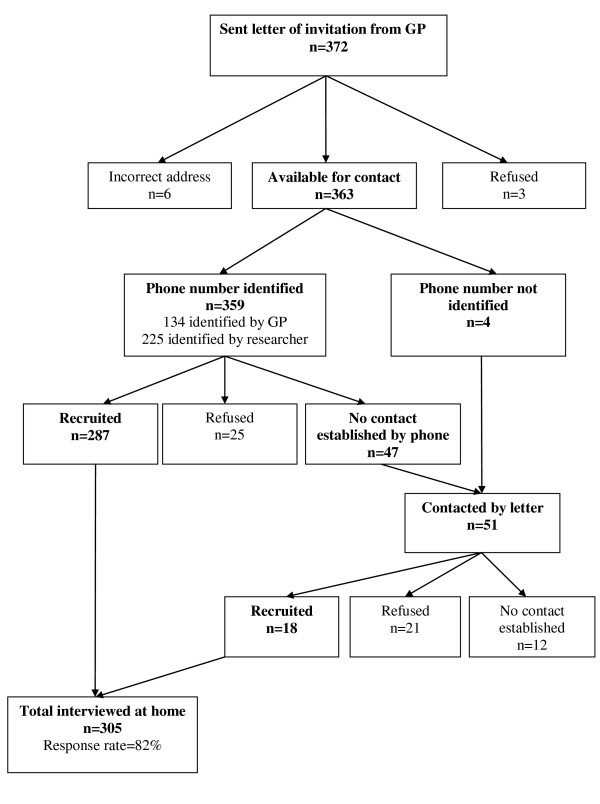
**Summary of recruitment**.

Figure 1 gives a summary of the recruitment process including the number of women contacted and the number recruited at each stage. Telephone numbers for 162 women were initially received from the GPs, but only 134 of these were valid (ie rang when dialled). Searching of the web-based directories yielded another 187 numbers. Further searching of GP records by practice staff provided 31 more. Finally health visitors (nurses trained in health promotion) were able to supply an additional 7 numbers. In total valid telephone numbers (ie number rang when dialled) were obtained for 359 of the 363 women with valid addresses.

Of the 305 women interviewed, 287 were recruited by a telephone call from the researcher (Table 1). Sixty women (16.7% of those telephoned) were recruited at the first telephone call. The recruitment rate increased to the fourth attempt when 57.4% of those telephoned agreed to take part. The number recruited on the fifth and sixth telephone calls was low (7.9% and 3.8% of those contacted). The overall refusal rate was low but was slightly higher for the later contacts. Overall, only 7% of those contacted by telephone refused to be interviewed.

**Table 1:** Recruitment rate at successive telephone calls

Phone call*	Number telephoned	Number of calls yielding interviews	Refused
1	359	60 (16.7%)	2 (0.6%)
2	297	46 (15.5%)	4 (1.3%)
3	247	81 (32.8%)	4 (1.6%)
4	162	93 (57.4%)	6 (3.7%)
5	63	5 (7.9%)	5 (7.9%)
6	53	2 (3.8%)	4 (7.5%)

* When a woman refused to participate no further contact attempts were made

The 47 women who did not answer the telephone calls, plus four for whom telephone numbers were unavailable, were contacted by letter. Twenty two women responded to this letter by calling the researcher. Twelve of these women were subsequently recruited and ten refused to take part. The 29 women who did not respond to this letter were sent a second letter, which gave a specific appointment time for a home visit. Thirteen women were at home at the time of the interview of whom six were recruited. Visiting cards were left at the homes of the 16 women who were out at the first home visit, offering a new appointment time. Of the 16, only four were at home at the second visit and all of them refused to participate.

Overall, of the 372 women invited to take part, 305 were recruited giving a recruitment rate of 82%. Most of the appointments for home interview were arranged by the telephone contact (Table 2). However the yield for each method of contact (ie the percent of those contacted who were subsequently interviewed) exceeded 20% for all methods except the final home visit. Only 49 women refused to take part; 3 at the initial invitation letter, 25 at telephone contact, 10 at the postal contact and 11 at the home visits. As might be expected the refusal rate progressively increased as the contact method was switched from telephone to postal approach to home visit (Table 2).

**Table 2:** Recruitment rate by method of approach

Method of approach	Number approached	Interviewed n (%*)	Refused n (%*)	Not contacted n (%*)
Invitation letter	372	n/a	3 (1.0)	6 (2.0)
Telephone	359	287 (79.9)	25 (7.0)	47 (13.1)
Letter seeking contact	51	12 (23.5)	10 (19.6)	29 (56.9)
First home visit	29	6 (20.7)	7 (24.1)	16 (55.2)
Second home visit	16	0 (0)	4 (25.0)	12 (75.0)

* Percentages were calculated using all those contacted by this method as the denominator

The non-contact rate in this study was very low. At the end of all the contact procedures only 18 women (5%) were not contacted by the researcher: six for whom the initial address was known to be incorrect and 12 who remained non-contactable after the final home visit. The non-contact rate increased dramatically with successive methods of contact (Table 2).

## Discussion

This study achieved a very high response among people living in deprived areas, a group that are known to be difficult to recruit to research studies [4,13-15]. The sequence of letter of invitation, followed by up to six attempts for a telephone contact, and finally resorting to postal approaches used, resulted in a very low non-contact rate. Telephone numbers were obtained for almost all of the potential participants. The refusal rate at telephone contact was very low, possibly because the researcher was able to talk to potential participants before recruitment. The researcher was able to form a rapport, answer any queries about the study and arrange appointments for interviews at a time that suited the mothers, including evenings and weekends. Thus making contact by telephone offers the benefits of a high contact rate and a low refusal rate.

When attempts to make contact by telephone were unsuccessful, postal invitations were used. Trials comparing the effect of contact sequence found that telephone then postal contact gives a higher response than the reverse [22,23]. In this study, because the target group were mothers of young children we anticipated a high level of telephone ownership. Further most telephones have a voice message facility which enables contact to be established even when the telephone is not answered. A key finding of this study is that recruitment rates remained high up to the fourth attempt at telephone contact. As this is a relatively inexpensive method of approach, multiple attempts at contact are strongly recommended.

A crucial requirement for high response rates is accurate contact details. Thus several strategies were used to obtain telephone numbers, to reduce the potential non-response bias. All types of approach (initial data from GPs, web based searches and further contact with practice staff) proved fruitful. The web-based searches were carried out early to reduce the workload for practice staff as much as possible. However this transfers the burden to the researcher, as the process of gaining access to websites and the conduct of trial and error searches was time-consuming. The very high contact rate achieved in this study indicates the value of using multiple sources to identify telephone numbers. The use of multiple sources also reduces the opportunity for selection bias that could arise if only one or two sources were used.

Gaining access to some web-based directories required the researcher to provide an e-mail address, a telephone number or personal details. Clearly some researchers may be reluctant to give personal details because this might lead to unsolicited contacts. However this can be easily overcome by providing dedicated mobile telephones and e-mail addresses for the duration of a study. A further problem is that some websites charge for use. These costs are likely to be modest in comparison with the total study costs, although researchers could take advantage of offers of free trials.

Making an approach by telephone might raise concerns in respondents' minds about how the telephone number was obtained. However the use of telephone surveys is a common practice[14,24,25] and telephone follow-up of non-responders has been used to increase response rates [23,26]. In this study the refusal rate at each telephone contact was small suggesting that there was little concern at being contacted. A previous study also found low levels of concern about telephone contact [27]. The lack of concern in the present study may have occurred because the invitation letter informed the women that they would be approached and gave them the opportunity to opt out. Another factor could be that unsolicited telephone contact by commercial organisations (tele-marketing) is now commonplace. Although these arguments seem plausible, telephone contact could raise ethical issues. People may be unaware that their telephone numbers, including mobile telephone numbers, could be found on websites. Thus unexpected calls could raise concerns about privacy. Further research on attitudes to contact by telephone would seem warranted.

Another factor which might have helped increase the interview rate was the interest of the topic to the mothers. Although we did not collect data on this we suspect that many mothers of young children would enjoy discussing their child with others. There is good evidence that saliency, the perceived importance of the topic, has a strong effect on response rates [28].

Monetary incentives were not used to increase recruitment in this study. Participants were offered a €10 gift voucher on completion of the study. Thus, the gift voucher was not used as an incentive to recruitment but as a reward for taking part. An even higher response rate may have been obtained if the €10 was offered to potential participants in the letter of invitation or during telephone contact with the researcher.

A technique which we did not use, refusal conversion, is common in national surveys [2,3,5]. In this, those who initially refuse are approached by expert interviewers who attempt to persuade them to participate. Conversion attempts can be successful with up to 40% of refusers [29] and thus could have a considerable impact on non-response. Whether such methods would be acceptable to the ethics committees that are important feature of medical research, remains open to question.

A limitation of the study is that it was not designed to answer the question: was the final recruitment rate worth the cost of the effort involved? This question needs to be looked at explicitly by investigating costs using a different research design. Several other strategies have already been tested in randomised controlled trials eg financial incentives, hand signed letters, succinct questionnaires and the use of reminders[6,7,11].

## Conclusion

This study has found that a high response rate can be obtained for a hard to reach group. This was achieved by making multiple efforts to obtain accurate contact details, together with repeated efforts at contact. The strategy of using telephone as the initial method of contact, although little used at present, may have considerable value for home-based interview surveys.

## Competing interests

The authors declare that they have no competing interests.

## Authors' contributions

The initial survey was conceived by IKC and designed by IKC, LI, VS, KP, WLW, PWS. KK developed the methods to obtain telephone numbers and made all the attempts at patient contact. IKC, KK and LI conducted the analyses and wrote the initial draft of the manuscript. All authors then contributed to produce the final manuscript.

## Pre-publication history

The pre-publication history for this paper can be accessed here:

http://www.biomedcentral.com/1471-2288/9/46/prepub
